# Supplementary material for: Dynamic estimation of auditory temporal response functions via state-space models with Gaussian mixture process noise
Source: PLoS Comput Biol. 2020 Aug 19;16(8):e1008172. doi: 10.1371/journal.pcbi.1008172 (PMC7485982; doi:10.1371/journal.pcbi.1008172)
Supplement: S1 Appendix — This appendix includes: (i) detailed derivations of the two approaches used for computing the expectations in Eqs (9), (10), and (11), (ii) the criteria for model order selection, and (iii) details of the MSAR estimation procedure. (PDF) [file pcbi.1008172.s001.pdf]

S1 Appendix
Supplementary Methods for "Dynamic Estimation of Auditory Temporal Response Functions via State-Space Models with Gaussian Mixture Process Noise"

Sina Miran[1], Alessandro Presacco[2], Jonathan Z. Simon[2,3,4], Michael C. Fu[2,5], Steven I. Marcus[2,3], Behtash Babadi[2,3,*]

1 Starkey Hearing Technologies, Eden Prairie, MN, USA

2 Institute for Systems Research, University of Maryland, College Park, MD, USA

3 Department of Electrical & Computer Engineering, University of Maryland, College Park, MD, USA

4 Department of Biology, University of Maryland, College Park, MD, USA

5 Robert H. Smith School of Business, University of Maryland, College Park, MD, USA

* behtash@umd.edu

In this appendix, we provide: (i) detailed derivations of the two approaches used for computing the expectations in Eqs. (9), (10), and (11), (ii) the criteria for model order selection, and (iii) details of the MSAR estimation procedure. In what follows, we use the notation $\boldsymbol{\mathcal{Y}}_{n_1}^{n_2}$ to denote the set of observations from n_1 to n_2, i.e., $\mathbf{y}_{n_1:n_2}$, and similarly define $\boldsymbol{\mathcal{X}}_{n_1}^{n_2}$ and $\mathcal{Z}_{i_1}^{i_2}$ for $\mathbf{x}_{n_1:n_2}$ and $z_{i_1:i_2}$, respectively.

Approach 1: Monte Carlo Approximations

One way to approximate the expectations in the update equations of the M-step is to utilize Monte Carlo methods. Let $\mathbf{x}_{(i,0):(i,W)}^{(u)}$ for $u = 1, \ldots, U$ denote a total of U sample paths, i.e., particles, with corresponding weights of $\omega_i^{(u)}$ inside the ith window to approximate the joint smoothing density of $\boldsymbol{\mathcal{X}}_{(i,0)}^{(i,W)} \mid \boldsymbol{\mathcal{Y}}_1^N, \widehat{\Theta}^{(\ell)}$ for $i = 1, \ldots, K$. Using this particle approximation, the update equations of the M-step become:

$$\widehat{p}_m^{(\ell+1)} = \frac{1}{K}\sum_{i=1}^{K}\sum_{u=1}^{U}\omega_i^{(u)}\widehat{\epsilon}_{i,m}^{(\ell,u)}, \tag{S1}$$

$$\widehat{\boldsymbol{\mu}}_m^{(\ell+1)} = \frac{\sum_{i=1}^{K}\sum_{u=1}^{U}\omega_i^{(u)}\widehat{\epsilon}_{i,m}^{(\ell,u)}\sum_{j=1}^{W}\mathbf{v}_{(i,j)}^{(u)}}{W\sum_{i=1}^{K}\sum_{u=1}^{U}\omega_i^{(u)}\widehat{\epsilon}_{i,m}^{(\ell,u)}}, \tag{S2}$$

$$\widehat{\boldsymbol{\Sigma}}_m^{(\ell+1)} = \frac{\sum_{i=1}^{K}\sum_{u=1}^{U}\omega_i^{(u)}\widehat{\epsilon}_{i,m}^{(\ell,u)}\sum_{j=1}^{W}\left(\mathbf{v}_{(i,j)}^{(u)} - \widehat{\boldsymbol{\mu}}_m^{(\ell+1)}\right)\left(\mathbf{v}_{(i,j)}^{(u)} - \widehat{\boldsymbol{\mu}}_m^{(\ell+1)}\right)^{\top}}{W\sum_{i=1}^{K}\sum_{u=1}^{U}\omega_i^{(u)}\widehat{\epsilon}_{i,m}^{(\ell,u)}}, \tag{S3}$$

where $\mathbf{v}_{(i,j)}^{(u)} = \mathbf{x}_{(i,j)}^{(u)} - f_{(i,j)}\left(\mathbf{x}_{(i,j\text{-}1)}^{(u)}\right)$, and $\widehat{\epsilon}_{i,m}^{(\ell,u)}$ is defined similarly to $\widehat{\epsilon}_{i,m}^{(\ell)}$ in (8) with $\widehat{\pi}_{(i,j),m}^{(\ell)}$'s evaluated at $\boldsymbol{\mathcal{X}}_{(i,0)}^{(i,W)} = \mathbf{x}_{(i,0):(i,W)}^{(u)}$ and $\Theta = \widehat{\Theta}^{(\ell)}$ in Eq. (6). Particle smoothing approaches are SMC methods that provide the sample paths $\mathbf{x}_{(i,0):(i,W)}^{(u)}$ and their respective weights $\omega_i^{(u)}$ [1]. The class of algorithms using SMC within EM for SSMs are referred to as SMCEM [2]. A forward-backward particle smoothing algorithm is presented in Algorithm S1 as an example of how the approximating particles can be computed.

Remark 1. In general, particle smoothing approaches are computationally intensive, especially for high-dimensional problems, which limits their application compared to

Algorithm S1. A Forward-Backward Particle Smoothing Alg.

Input: state-space model in (1) and parameter estimate $\widehat{\Theta}^{(\ell)}$.
Output: sample paths $\mathbf{x}_{(i,0):(i,W)}^{(u)}$ and their weights $\omega_i^{(u)}$.

1: Initialize $\mathbf{x}_0^{(u)}$ and their filtering weights $\bar{\omega}_0^{(u)} = 1/U$.
2: **for** $i = 1 : K$ **do**
3: Sample $z_i^{(u)}$ according to $\widehat{p}_{1:M}^{(\ell)}$.
4: Sample $\mathbf{x}_{(i,0):(i,W)}^{(u)}$ using $\mathbf{x}_{(i,0)}^{(u)}$ as the starting point and $z_i^{(u)}$ as the active Gaussian component.
5: $\widetilde{\omega}_i^{(u)} = \prod_{j=1}^{W} \mathrm{P}\left(\mathbf{y}_{(i,j)}^{(u)} \,\middle|\, \mathbf{x}_{(i,j)}^{(u)}\right)$.
6: Normalize the weights such that $\sum_{u=1}^{U}\bar{\omega}_i^{(u)} = 1$.
7: Resample $\mathbf{x}_{(i,W)}^{(u)}$ for next window according to $\bar{\omega}_i^{(u)}$.
8: **end for**
9: Initialize the smoothing weights $\omega_K^{(u)} = \widetilde{\omega}_K^{(u)}$.
10: **for** $i = K-1 : 1$ **do**
11: $\omega_i^{(u)} = \widetilde{\omega}_i^{(u)} \sum_{u'=1}^{U} \frac{\mathrm{P}\left(\mathbf{x}_{(i+1,1)}^{(u')} \,\middle|\, \mathbf{x}_{(i,W)}^{(u)}, \widehat{\Theta}^{(\ell)}\right)\omega_{i+1}^{(u')}}{\sum_{u''=1}^{U}\mathrm{P}\left(\mathbf{x}_{(i+1,1)}^{(u')} \,\middle|\, \mathbf{x}_{(i,W)}^{(u'')}, \widehat{\Theta}^{(\ell)}\right)\widetilde{\omega}_i^{(u'')}}$.
12: **end for**

particle filtering methods. In our setting, densities of dimension $d_x(W+1)$ have to be approximated by particles. The forward-backward method in Algorithm S1 simply re-weights the filtering particles according to future observations and incurs an $\mathcal{O}(U^2)$ cost. The two-filter particle smoother [3] samples the particles in the smoothing step but has a similar computational cost. In [3], an approximation based on spatial-index methods is introduced to reduce the computational cost to $\mathcal{O}(U \log U)$. Finally, a particle smoothing method with $\mathcal{O}(U)$ cost (similar to that of particle filtering) is developed in [4]. However, it operates under the assumption of minimal posterior dependence between $\mathbf{x}_{n-1}$ and $\mathbf{x}_{n+1}$ when sampling for the smoothing density of $\mathbf{x}_n$.

Approach 2: Closed-Form Approximations

In this section, we consider a linear SSM, i.e., $f_n(\mathbf{x}_{n-1}) = \mathbf{A}_n \mathbf{x}_{n-1}$ and $g_n(\mathbf{x}_n) = \mathbf{C}_n \mathbf{x}_n$ in (1), to exploit the GM formulation of the smoothing densities [5]. Techniques such as the extended Kalman filter [6] or the unscented Kalman filter [7] are often used to approximate the general state-space model of Eq. (1) with a linear model. We introduce an approximation to the expectations in the M-step which allows to employ GM smoothing densities for computing the updated parameters in EM. This is akin to the application of EM in linear Gaussian SSMs [8]. Then, we construct an algorithm to efficiently compute the required smoothing densities in closed-form for our setting. As a result, the computational cost of the M-step would be comparable to performing parallel instances of fixed-interval smoothing, each corresponding to a component of the GM process noise.

We first consider a 0th-order Taylor expansion for $\widehat{\epsilon}_{i,m}^{(\ell)}$ in the update formulas of (9)-(10) around the mean of the smoothing densities. In other words, $\widehat{\epsilon}_{i,m}^{(\ell)} \approx \bar{\epsilon}_{i,m}^{(\ell)}$ where $\bar{\epsilon}_{i,m}^{(\ell)}$ is computed similarly to (8) with $\widehat{\pi}_{(i,j),m}^{(\ell)}$'s evaluated at $\boldsymbol{\mathcal{X}}_1^N = \bar{\mathbf{x}}_{1:N} := \mathrm{E}_{\boldsymbol{\mathcal{X}}}\{\mathbf{x}_{1:N}\}$ and $\Theta = \widehat{\Theta}^{(\ell)}$ in Eq. (6).

Remark 2. Note that this approximation is valid when the GM smoothing densities (over which the expectations are computed) do not exhibit multimodal behavior with mixture components far from each other. Otherwise, the 0th order approximation must be carried out at the mean of each mixture component separately (rather than at the mean of the smoothing density). Under high enough observation signal-to-noise ratio

(SNR), the GM smoothing densities are expected to mainly consist of mixture components with similar means, so the resulting density exhibits a unimodal morphology concentrated on the ML estimate of the states. Therefore, approximation of $\widehat{\epsilon}_{i,m}^{(\ell)}$ by its value at the mean of the smoothing density would not introduce significant error at high SNRs. It is worth noting that higher order approximations to $\widehat{\epsilon}_{i,m}^{(\ell)}$ can be considered at the cost of more computational cost, which would also result in higher moments of GM smoothing densities appearing in the M-step update equations. As we will demonstrate in the Results section, the 0th order approximation suffices for our applications.

It is known that for a linear SSM with Gaussian mixture noise, the filtering and smoothing densities also take Gaussian mixture forms [5,9]. Let

$$\mathrm{P}\left(\boldsymbol{\mathcal{X}}_{n-1}^{n} \,\middle|\, \boldsymbol{\mathcal{Y}}_{1}^{N}, \widehat{\Theta}^{(\ell)}\right) = \sum_{\gamma=1}^{\Gamma_{\mathsf{S}}} \rho_n^{(\mathsf{s},\gamma)} \mathcal{N}\left(\begin{bmatrix}\mathbf{x}_{n-1}\\ \mathbf{x}_n\end{bmatrix}; \boldsymbol{\mu}_n^{(\mathsf{s},\gamma)}, \boldsymbol{\Sigma}_n^{(\mathsf{s},\gamma)}\right) \tag{S4}$$

be the one-step joint smoothing density at time n, where the superscript s identifies smoothing parameters, and Γ_{S} is the number of mixture components forming the smoothing density. Taking $\bar{\epsilon}_{i,m}^{(\ell)}$ out of the expectations, the M-step update equations become:

$$\widehat{p}_m^{(\ell+1)} = \frac{1}{K}\sum_{i=1}^{K} \bar{\epsilon}_{i,m}^{(\ell)}, \quad \widehat{\boldsymbol{\mu}}_m^{(\ell+1)} = \frac{\sum_{i=1}^{K} \bar{\epsilon}_{i,m}^{(\ell)} \sum_{j=1}^{W} \widetilde{\mathbf{A}}_{(i,j)} \sum_{\gamma=1}^{\Gamma_{\mathsf{S}}} \rho_{(i,j)}^{(\mathsf{s},\gamma)} \boldsymbol{\mu}_{(i,j)}^{(\mathsf{s},\gamma)}}{W \sum_{i=1}^{K} \bar{\epsilon}_{i,m}^{(\ell)}},$$

$$\widehat{\boldsymbol{\Sigma}}_m^{(\ell+1)} = \frac{\sum_{i=1}^{K} \bar{\epsilon}_{i,m}^{(\ell)} \sum_{j=1}^{W} \widetilde{\mathbf{A}}_{(i,j)} \sum_{\gamma=1}^{\Gamma_{\mathsf{S}}} \rho_{(i,j)}^{(\mathsf{s},\gamma)} \left(\boldsymbol{\Sigma}_{(i,j)}^{(\mathsf{s},\gamma)} + \boldsymbol{\mu}_{(i,j)}^{(s,\gamma)} \left(\boldsymbol{\mu}_{(i,j)}^{(s,\gamma)}\right)^{\top}\right) \widetilde{\mathbf{A}}_{(i,j)}^{\top}}{W \sum_{i=1}^{K} \bar{\epsilon}_{i,m}^{(\ell)}} - \widehat{\boldsymbol{\mu}}_m^{(\ell+1)} \widehat{\boldsymbol{\mu}}_m^{(\ell+1)\top}, \tag{S5}$$

where $\widetilde{\mathbf{A}}_{(i,j)} = [\text{-}\mathbf{A}_{(i,j)}, \mathbf{I}_{d_x}]$ with $\mathbf{I}_{d_x}$ denoting the identity matrix of dimension d_x. Note that the update rule for $\mathbf{R}$ in Eq. (11) takes the form:

$$\widehat{\mathbf{R}}^{(\ell+1)} = \frac{1}{N}\sum_{n=1}^{N} \mathbf{y}_n \mathbf{y}_n^{\top} - \sum_{i=1}^{K}\sum_{m=1}^{M} \bar{\epsilon}_{i,m}^{(\ell)} \sum_{j=1}^{W}\sum_{\gamma=1}^{\Gamma_{\mathsf{S}}} \left\{ \mathbf{y}_{(i,j)} \left(\boldsymbol{\mu}_{(i,j)}^{(s,\gamma)}\right)_2^{\top} \mathbf{C}_n^{\top} + \mathbf{C}_n \left(\boldsymbol{\mu}_{(i,j)}^{(s,\gamma)}\right)_2 \mathbf{y}_n^{\top} - \mathbf{C}_n \left(\boldsymbol{\Sigma}_{(i,j)}^{(\mathsf{s},\gamma)}\right)_{2,2} \mathbf{C}_n^{\top} \right\}. \tag{S6}$$

Similarly, the coordinate descent update rule for α in Eq. (12) takes the form:

$$\widehat{\alpha}^{(\ell+1)} = \frac{\sum_{i=1}^{K}\sum_{m=1}^{M}\bar{\epsilon}_{i,m}^{(\ell)}\sum_{j=1}^{W}\sum_{\gamma=1}^{\Gamma_{\mathsf{S}}}\operatorname{Tr}\left\{\widehat{\mathbf{\Sigma}}_m^{(\ell+1)-1}\left(\mathbf{\Sigma}_{(i,j)}^{(\mathsf{s},\gamma)}\right)_{1,1}\right\}}{\sum_{i=1}^{K}\sum_{m=1}^{M}\bar{\epsilon}_{i,m}^{(\ell)}\sum_{j=1}^{W}\sum_{\gamma=1}^{\Gamma_{\mathsf{S}}}\operatorname{Tr}\left\{\widehat{\mathbf{\Sigma}}_m^{(\ell+1)-1}\left(\left(\mathbf{\Sigma}_{(i,j)}^{(\mathsf{s},\gamma)}\right)_{2,1}-\left(\boldsymbol{\mu}_{(i,j)}^{(\mathsf{s},\gamma)}\right)_1^{\top}\widehat{\boldsymbol{\mu}}_m^{(\ell+1)}\right)\right\}}. \quad \text{(S7)}$$

where $(\mathbf{Z})_{a,b}$ and $(\mathbf{z})_a$ denote the $(a,b)^{\mathsf{th}}$ block and a^{th} block of the matrix $\mathbf{Z}$ and vector $\mathbf{z}$, respectively. In the TRF model of Eq. (17), we have $\mathbf{C}_n = \mathbf{S}_n^{\top}\widetilde{\mathbf{G}}$.

Another approach to approximately compute the expectations in the update equations (9)-(10) is to use the Laplace approximation [10]. This approach, however, requires the computation of the GM joint smoothing density of $\boldsymbol{\mathcal{X}}_{(i,0)}^{(i,W)} \mid \boldsymbol{\mathcal{Y}}_1^N, \widehat{\Theta}^{(\ell)}$ and is more computationally intensive than the current approximation, which only requires the one-step smoothing covariances regardless of the choice of W.

The smoothing density parameters in Eq. (S4), i.e., $\left\{\rho_n^{(\mathsf{s},\gamma)}, \boldsymbol{\mu}_n^{(\mathsf{s},\gamma)}, \mathbf{\Sigma}_n^{(\mathsf{s},\gamma)}\right\}$, have to be estimated for $n = 1, \ldots, N$ in the E-step. In Section II.D of [5], a forward-backward recursion is used to obtain closed-form solutions for smoothing densities under a linear SSM with GM noise components. The dimension of the underlying matrices and matrix inversion costs, however, grows with n as the recursions proceed, which limits the utility of the algorithm for practical applications even with moderate observation duration. In [9], the two-filter formula is adopted to compute the GM smoothing densities by transforming the smoothing problem to a filtering one. An underlying assumption in [9] is that either $\mathbf{C}_n$ is invertible or consecutive observations can be concatenated such that the effective measurement matrix is invertible. As this assumption does not hold in general, we instead develop a recursive algorithm based on the two-filter formula in our setting to compute the smoothing parameters in (S4) in closed-form. Since all of the following densities are conditioned on $\widehat{\Theta}^{(\ell)}$ similar to Eq. (S4), we hereafter drop the conditioning in our notation for convenience.

Let the filtering density at the end of the $(i-1)^{\text{st}}$ window be

$$\mathrm{P}\left(\mathbf{x}_{(i,0)} \,\middle|\, \boldsymbol{\mathcal{Y}}_1^{(i,0)}\right) = \sum_{\gamma=1}^{\Gamma_{\mathsf{F}}} \rho_{(i,0)}^{(\mathsf{f},\gamma)} \mathcal{N}\left(\mathbf{x}_{(i,0)}; \boldsymbol{\mu}_{(i,0)}^{(\mathsf{f},\gamma)}, \mathbf{\Sigma}_{(i,0)}^{(\mathsf{f},\gamma)}\right), \quad \text{(S8)}$$

where superscript f identifies forward filtering parameters and Γ_{F} is the number of mixtures forming the filtering density at the end of each window. Also, let the unnormalized backward information filter [9] at the end of the ith window be

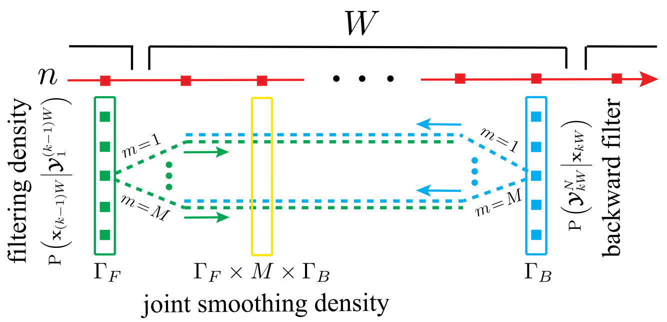

Fig S1. Schematic depiction of forward/backward updates for Eq. (S10).

$$\mathrm{P}\left(\boldsymbol{\mathcal{Y}}_{iW}^{N}\,\middle|\,\mathbf{x}_{iW}\right) \propto \sum_{\gamma=1}^{\Gamma_\mathsf{B}} \beta_{iW}^{(\gamma)} \exp\left\{-\frac{1}{2}\mathbf{x}_{iW}^\top \mathbf{B}_{iW}^{(\gamma)}\mathbf{x}_{iW} + \mathbf{x}_{iW}^\top \mathbf{b}_{iW}^{(\gamma)}\right\}, \tag{S9}$$

where Γ_B is the number of exponential components forming the information filter at the end of each window. Note that Eq. (S9) is not a density in $\mathbf{x}$. Considering the independence of z_i and $\boldsymbol{\mathcal{Y}}_1^{(i,0)}$, the two-filter formula for window i in our switching GM process noise model can be written as

$$\mathrm{P}\left(\boldsymbol{\mathcal{X}}_{(i,j\text{-}1)}^{(i,j)}\,\middle|\,\boldsymbol{\mathcal{Y}}_1^{N}\right) = \frac{1}{\mathrm{P}\left(\boldsymbol{\mathcal{Y}}_{(i,1)}^{N}\,\middle|\,\boldsymbol{\mathcal{Y}}_1^{(i,0)}\right)} \sum_{m=1}^{M} \widehat{p}_m^{(\ell)}\,\mathrm{P}\left(\mathbf{x}_{(i,j\text{-}1)}, \boldsymbol{\mathcal{Y}}_{(i,1)}^{(i,j\text{-}1)}\,\middle|\,\boldsymbol{\mathcal{Y}}_1^{(i,0)}, \mathfrak{z}_i^m\right) \times \\ \mathrm{P}\left(\mathbf{x}_{(i,j)}\,\middle|\,\mathbf{x}_{(i,j\text{-}1)}, \mathfrak{z}_i^m\right)\mathrm{P}\left(\boldsymbol{\mathcal{Y}}_{(i,j)}^{N}\,\middle|\,\mathbf{x}_{(i,j)}, \mathfrak{z}_i^m\right), \tag{S10}$$

where $\mathfrak{z}_i^m$ stands for the event $\{z_i = m\}$. Our main objective here is to find a recursive update rule for the joint density of Eq. (S10). To this end, we derive recursive formulas for propagating the forward and backward filters, i.e., the components in the summand of Eq. (S10), within each window (See Fig. S1 for a schematic depiction of this procedure).

The leftmost term in the summand of Eq. (S10) is the forward filter and represents an unnormalized filtering density, which we express as:

$$\mathrm{P}\left(\mathbf{x}_{(i,j)}, \boldsymbol{\mathcal{Y}}_{(i,1)}^{(i,j)}\,\middle|\,\boldsymbol{\mathcal{Y}}_1^{(i,0)}, \mathfrak{z}_i^m\right) = \sum_{\gamma=1}^{\Gamma_\mathsf{F}} \rho_{(i,j),m}^{(\mathsf{f},\gamma)}\,\mathcal{N}\left(\mathbf{x}_{(i,j)}; \boldsymbol{\mu}_{(i,j),m}^{(\mathsf{f},\gamma)}, \boldsymbol{\Sigma}_{(i,j),m}^{(\mathsf{f},\gamma)}\right) \tag{S11}$$

for $j = 1, \ldots, W$ and compute it through the following unnormalized forward recursion in j:

$$\mathrm{P}\left(\mathbf{x}_{(i,j)}, \boldsymbol{\mathcal{Y}}_{(i,1)}^{(i,j)} \,\middle|\, \boldsymbol{\mathcal{Y}}_{1}^{(i,0)}, \mathfrak{z}_i^m\right) = \int \mathrm{P}\left(\mathbf{y}_{(i,j)} \mid \mathbf{x}_{(i,j)}\right) \mathrm{P}\left(\mathbf{x}_{(i,j)} \mid \mathbf{x}_{(i,j\text{-}1)}, \mathfrak{z}_i^m\right) \times$$
$$\mathrm{P}\left(\mathbf{x}_{(i,j\text{-}1)}, \boldsymbol{\mathcal{Y}}_{(i,1)}^{(i,j\text{-}1)} \,\middle|\, \boldsymbol{\mathcal{Y}}_{1}^{(i,0)}, \mathfrak{z}_i^m\right) d\mathbf{x}_{(i,j\text{-}1)}.$$

The recursion is initialized by the filtering density in Eq. (S8) at window i. This results in the following forward filter parameter updates:

$$\begin{cases} \widetilde{\boldsymbol{\mu}} = \mathbf{A}_{(i,j)} \boldsymbol{\mu}_{(i,j\text{-}1),m}^{(\mathsf{f},\gamma)} + \widehat{\boldsymbol{\mu}}_m^{(\ell)} \\ \widetilde{\boldsymbol{\Sigma}} = \mathbf{A}_{(i,j)} \boldsymbol{\Sigma}_{(i,j\text{-}1),m}^{(\mathsf{f},\gamma)} \mathbf{A}_{(i,j)}^\top + \widehat{\boldsymbol{\Sigma}}_m^{(\ell)} \\ \mathbf{H} = \widetilde{\boldsymbol{\Sigma}} \mathbf{C}_{(i,j)}^\top \left(\mathbf{C}_{(i,j)} \widetilde{\boldsymbol{\Sigma}} \mathbf{C}_{(i,j)}^\top + \mathbf{R} \right)^{-1} \\ \boldsymbol{\mu}_{(i,j),m}^{(\mathsf{f},\gamma)} = \widetilde{\boldsymbol{\mu}} + \mathbf{H} \left(\mathbf{y}_{(i,j)} - \mathbf{C}_{(i,j)} \widetilde{\boldsymbol{\mu}} \right) \\ \boldsymbol{\Sigma}_{(i,j),m}^{(\mathsf{f},\gamma)} = \left(\mathbf{I} - \mathbf{H} \mathbf{C}_{(i,j)} \right) \widetilde{\boldsymbol{\Sigma}} \\ \rho_{(i,j),m}^{(\mathsf{f},\gamma)} = \rho_{(i,j\text{-}1),m}^{(\mathsf{f},\gamma)} \mathcal{N}\left(\mathbf{y}_{(i,j)}; \mathbf{C}_{(i,j)} \widetilde{\boldsymbol{\mu}}, \mathbf{C}_{(i,j)} \widetilde{\boldsymbol{\Sigma}} \mathbf{C}_{(i,j)}^\top + \mathbf{R} \right) \end{cases} \tag{S12}$$

and the filtering density at time (i, j) is computed as

$$\mathrm{P}\left(\mathbf{x}_{(i,j)} \,\middle|\, \boldsymbol{\mathcal{Y}}_{1}^{(i,j)}\right) \propto \sum_{m=1}^{M} \widehat{p}_m \, \mathrm{P}\left(\mathbf{x}_{(i,j)}, \boldsymbol{\mathcal{Y}}_{(i,1)}^{(i,j)} \,\middle|\, \boldsymbol{\mathcal{Y}}_{1}^{(i,0)}, \mathfrak{z}_i^m\right). \tag{S13}$$

Next, we represent the unnormalized backward information filter $\mathrm{P}\left(\boldsymbol{\mathcal{Y}}_{(i,j)}^N \mid \mathbf{x}_{(i,j)}, \mathfrak{z}_i^m\right)$ in Eq. (S10), as

$$\sum_{\gamma=1}^{\Gamma_{\mathsf{B}}} \beta_{(i,j),m}^{(\gamma)} \exp\left\{ -\frac{1}{2} \mathbf{x}_{(i,j)}^\top \mathbf{B}_{(i,j),m}^{(\gamma)} \mathbf{x}_{(i,j)} + \mathbf{x}_{(i,j)}^\top \mathbf{b}_{(i,j),m}^{(\gamma)} \right\}, \tag{S14}$$

where we enforce the normalization $\sum_{\gamma=1}^{\Gamma_{\mathsf{B}}} \sum_{m=1}^{M} \beta_{(i,j),m}^{(\gamma)} = 1$. Note that this normalization is applied to avoid numerical instabilities while performing the recursions and does not change the final smoothing density of Eq. (S10), which has to be eventually normalized. The backward filter in Eq. (S14) can be computed through the following recursion [9]:

$$\mathrm{P}\left(\boldsymbol{\mathcal{Y}}_{(i,j)}^N \,\middle|\, \mathbf{x}_{(i,j)}, \mathfrak{z}_i^m\right) = \int \mathrm{P}\left(\mathbf{y}_{(i,j)} \mid \mathbf{x}_{(i,j)}\right) \mathrm{P}\left(\mathbf{x}_{(i,j+1)} \mid \mathbf{x}_{(i,j)}, \mathfrak{z}_i^m\right) \times$$
$$\mathrm{P}\left(\boldsymbol{\mathcal{Y}}_{(i,j+1)}^N \,\middle|\, \mathbf{x}_{(i,j+1)}, \mathfrak{z}_i^m\right) d\mathbf{x}_{(i,j+1)} \tag{S15}$$

and is initialized by Eq. (S9) at the end of window i. This results in the following backward filter parameter updates:

$$\begin{cases} \overline{\boldsymbol{\Sigma}} = \widehat{\boldsymbol{\Sigma}}_m^{(\ell)} \left(\mathbf{I} + \mathbf{B}_{(i,j+1),m}^{(\gamma)} \widehat{\boldsymbol{\Sigma}}_m^{(\ell)} \right) \\ \overline{\boldsymbol{\mu}} = \widehat{\boldsymbol{\Sigma}}_m^{(\ell)} \mathbf{b}_{(i,j+1),m}^{(\gamma)} + \widehat{\boldsymbol{\mu}}_m^{(\ell)} \\ \mathbf{B}_{(i,j),m}^{(\gamma)} = \mathbf{C}_{(i,j)}^{\top} \mathbf{R}^{-1} \mathbf{C}_{(i,j)} + \mathbf{A}_{(i,j+1)}^{\top} \left[\left(\widehat{\boldsymbol{\Sigma}}_m^{(\ell)} \right)^{-1} - \overline{\boldsymbol{\Sigma}}^{-1} \right] \mathbf{A}_{(i,j+1)} \\ \mathbf{b}_{(i,j),m}^{(\gamma)} = \mathbf{C}_{(i,j)}^{\top} \mathbf{R}^{-1} \mathbf{y}_{(i,j)} - \mathbf{A}_{(i,j+1)}^{\top} \left(\widehat{\boldsymbol{\Sigma}}_m^{(\ell)} \right)^{-1} \widehat{\boldsymbol{\mu}}_m^{(\ell)} + \mathbf{A}_{(i,j+1)}^{\top} \overline{\boldsymbol{\Sigma}}^{-1} \overline{\boldsymbol{\mu}} \\ \beta_{(i,j),m}^{(\gamma)} \propto \beta_{(i,j+1),m}^{(\gamma)} \sqrt{\frac{|\widehat{\boldsymbol{\Sigma}}_m^{(\ell)}|}{|\overline{\boldsymbol{\Sigma}}|}} \exp\left\{ -\frac{1}{2} \left(\widehat{\boldsymbol{\mu}}_m^{(\ell)} \right)^{\top} \left(\widehat{\boldsymbol{\Sigma}}_m^{(\ell)} \right)^{-1} \widehat{\boldsymbol{\mu}}_m^{(\ell)} + \frac{1}{2} \overline{\boldsymbol{\mu}}^{\top} \overline{\boldsymbol{\Sigma}}^{-1} \overline{\boldsymbol{\mu}} \right\} \end{cases} \tag{S16}$$

and the overall backward filter in the beginning of window i can be computed from Eq. (S14) as

$$\mathrm{P}\left(\boldsymbol{\mathcal{Y}}_{(i,0)}^{N} \,\middle|\, \mathbf{x}_{(i,0)} \right) = \sum_{m=1}^{M} \widehat{p}_M^{(\ell)} \, \mathrm{P}\left(\boldsymbol{\mathcal{Y}}_{(i,0)}^{N} \,\middle|\, \mathbf{x}_{(i,0)}, \mathfrak{z}_i^m \right). \tag{S17}$$

Using Eqs. (S11) and (S14), the parameters of the joint GM smoothing density in Eq. (S10) are computed as:

$$\begin{cases} \gamma'' = (\gamma - 1) M \Gamma_{\mathsf{B}} + (m-1)\Gamma_{\mathsf{B}} + \gamma' \\ \mathbf{S}_{11} = \left(\boldsymbol{\Sigma}_{(i,j-1),\gamma}^{(\mathsf{f},m)} \right)^{-1} + \mathbf{A}_{(i,j)}^{\top} \left(\widehat{\boldsymbol{\Sigma}}_m^{(\ell)} \right)^{-1} \mathbf{A}_{(i,j)} \\ \mathbf{S}_{12} = \mathbf{S}_{21}^{\top} = -\mathbf{A}_{(i,j)}^{\top} \left(\widehat{\boldsymbol{\Sigma}}_m^{(\ell)} \right)^{-1} \\ \mathbf{S}_{22} = \mathbf{B}_{(i,j),\gamma'}^{(m)} + \left(\widehat{\boldsymbol{\Sigma}}_m^{(\ell)} \right)^{-1} \\ \boldsymbol{\Sigma}_{(i,j),\gamma''}^{(\mathsf{s})} = \begin{bmatrix} \mathbf{S}_{11} & \mathbf{S}_{12} \\ \mathbf{S}_{21} & \mathbf{S}_{22} \end{bmatrix}^{-1} \\ \mathbf{u}_1 = \left(\boldsymbol{\Sigma}_{(i,j-1),\gamma}^{(\mathsf{f},m)} \right)^{-1} \boldsymbol{\mu}_{(i,j-1),\gamma}^{(\mathsf{f},m)} - \mathbf{A}_{(i,j)}^{\top} \left(\widehat{\boldsymbol{\Sigma}}_m^{(\ell)} \right)^{-1} \widehat{\boldsymbol{\mu}}_m^{(\ell)} \\ \mathbf{u}_2 = \left(\widehat{\boldsymbol{\Sigma}}_m^{(\ell)} \right)^{-1} \widehat{\boldsymbol{\mu}}_m^{(\ell)} + \mathbf{b}_{(i,j),\gamma'}^{(m)} \\ \boldsymbol{\mu}_{(i,j),\gamma''}^{(\mathsf{s})} = \boldsymbol{\Sigma}_{(i,j),\gamma''}^{(\mathsf{s})} \begin{bmatrix} \mathbf{u}_1 \\ \mathbf{u}_2 \end{bmatrix} \\ \rho_{(i,j),\gamma''}^{(\mathsf{s})} \propto \rho_{(i,j-1),\gamma}^{(\mathsf{f},m)} \widehat{p}_m^{(\ell)} \beta_{(i,j),\gamma'}^{(m)} \sqrt{\frac{|\boldsymbol{\Sigma}_{(i,j),\gamma''}^{(\mathsf{s})}|}{|\widehat{\boldsymbol{\Sigma}}_m^{(\ell)}| |\boldsymbol{\Sigma}_{(i,j-1),\gamma}^{(\mathsf{f},m)}|}} \\ \quad \times \exp\left\{ -\frac{1}{2} \left(\boldsymbol{\mu}_{(i,j-1),\gamma}^{(\mathsf{f},m)} \right)^{\top} \left(\boldsymbol{\Sigma}_{(i,j-1),\gamma}^{(\mathsf{f},m)} \right)^{-1} \boldsymbol{\mu}_{(i,j-1),\gamma}^{(\mathsf{f},m)} \right\} \\ \quad \times \exp\left\{ -\frac{1}{2} \left(\widehat{\boldsymbol{\mu}}_m^{(\ell)} \right)^{\top} \left(\widehat{\boldsymbol{\Sigma}}_m^{(\ell)} \right)^{-1} \widehat{\boldsymbol{\mu}}_m^{(\ell)} + \frac{1}{2} \left(\boldsymbol{\mu}_{(i,j),\gamma''}^{(\mathsf{s})} \right)^{\top} \left(\boldsymbol{\Sigma}_{(i,j),\gamma''}^{(\mathsf{s})} \right)^{-1} \boldsymbol{\mu}_{(i,j),\gamma''}^{(\mathsf{s})} \right\} \end{cases} \tag{S18}$$

where we have $\gamma \in \{1, \ldots, \Gamma_{\mathsf{F}}\}$, $m \in \{1, \ldots, M\}$, and $\gamma' \in \{1, \ldots, \Gamma_{\mathsf{B}}\}$. This brings the total number of mixture components in the joint smoothing density of Eq. (S10) to $\Gamma_{\mathsf{F}} \times M \times \Gamma_{\mathsf{B}}$. As the number of mixture components grows exponentially in SSMs with GM noise components [11], limiting them is a crucial step for practical purposes. To this end, in forming the density of Eq. (S4), the number of mixture components obtained from Eq. (S10) is reduced to Γ_{S} prior to updating the parameters in the M-step. In this work, we choose Γ_{S} components from the density of Eq. (S10) with the largest mixture probabilities for simplicity. However, more accurate mixture reduction algorithms are available and developed in [11–13], but with additional computational costs. It is worth noting that calculations corresponding to the weights $\rho^{(\mathsf{f})}$'s in Eq. (S12), β's in (S16), and $\rho^{(\mathsf{s})}$'s in (S18) should be performed in log-scale to avoid numerical errors in practice. Algorithm S2 summarizes the steps for calculating the smoothing density parameters in Eq. (S4) for $n = 1, \ldots, N$. Note that when the observation noise covariance matrix $\mathbf{R}$ is unknown, the smoothing densities of Eq. (S4) can be replaced in Eq. (11) to provide a closed-form update for $\widehat{\mathbf{R}}^{(\ell+1)}$.

Algorithm S2. Two-Filter Gaussian Mixture Smoothing Alg.

Input: linear state-space model in (1), parameter estimate $\widehat{\Theta}^{(\ell)}$, and component limits Γ_{F}, Γ_{B}, and Γ_{S}.

Output: smoothing density parameters $\rho^{(\mathsf{s})}_{n,\gamma}$, $\boldsymbol{\mu}^{(\mathsf{s})}_{n,\gamma}$, $\boldsymbol{\Sigma}^{(\mathsf{s})}_{n,\gamma}$ in (S4) for $n \in \{1, \ldots, N\}$ and $\gamma \in \{1, \ldots, \Gamma_{\mathsf{S}}\}$.

1: Initialize the filtering density in (S8) at $n = 0$ as the prior on $\mathbf{x}_0$.
2: **for** $i = 1 : K$ **do**
3: Run forward recursions of (S12) for $m = 1, \ldots, M$ in window i starting from (S8) and store the parameters.
4: Compute the filtering density at $n = iW$ from (S13).
5: Out of $\Gamma_{\mathsf{F}} \times M$ mixture components in the filtering density, keep Γ_{F} with the largest probabilities as initialization for window $i + 1$.
6: **end for**
7: Initialize the backward filter as $\mathrm{P}\left(\mathbf{y}_N \,\middle|\, \mathbf{x}_N\right)$, i.e., $\beta_{N,1} = 1$, $\mathbf{B}_{N,1} = \mathbf{C}_N^{\top}\mathbf{R}^{-1}\mathbf{C}_N$, and $\mathbf{b}_{N,1} = \mathbf{C}_N^{\top}\mathbf{R}^{-1}\mathbf{y}_N$.
8: **for** $i = K : 1$ **do**
9: Run backward recursions of (S16) for $m = 1, \ldots, M$ in window i starting from (S9).
10: Run smoothing algorithm of (S18) in window i using backward filtering parameters and the stored forward filtering parameters.
11: Out of $\Gamma_{\mathsf{F}} \times M \times \Gamma_{\mathsf{B}}$ smoothing mixture components, store Γ_{S} with the largest probabilities for smoothing densities of (S4) in window i.
12: Compute the backward filter at $n = (i-1)W$ from (S17).
13: Out of $\Gamma_{\mathsf{B}} \times M$ backward filtering components, keep Γ_{B} corresponding to the most significant mixture components of $\mathrm{P}\left(\boldsymbol{\mathcal{X}}^{(i,1)}_{(i,0)} \,\middle|\, \boldsymbol{\mathcal{Y}}_1^N\right)$ as initialization for window $i - 1$.
14: **end for**
15: Output the computed smoothing parameters of (S4).

Model Order Selection

An important issue in applications of GMs for clustering is the choice of the number of mixtures M. Various model selection criteria have been used in the literature of Gaussian mixtures, including the Akaike Information Criterion (AIC), Bayesian Information Criterion (BIC), and Integrated Completed Likelihood (ICL) [14–17], most of which require the computation of data log-likelihood. In Approach 1, the log-likelihood can be approximated using the unnormalized particle filtering weights $\widetilde{\omega}_i^{(u)}$'s in Algorithm S1 as

$$\log \mathrm{P}\left(\boldsymbol{\mathcal{Y}}_1^N\right) \approx \sum_{i=1}^K \log\left(\sum_{u=1}^U \widetilde{\omega}_i^{(u)}\right). \tag{S19}$$

In Approach 2, using the unnormalized filtering densities in closed-form approximation, the log-likelihood in our model can be computed based on [18] as

$$\begin{aligned}\log \mathrm{P}\left(\boldsymbol{\mathcal{Y}}_1^N\right) &= \sum_{i=1}^K \log\left(\sum_{m=1}^M \widehat{p}_m^{(\ell)}\, \mathrm{P}\left(\boldsymbol{\mathcal{Y}}_{(i,1)}^{(i,W)} \,\middle|\, \boldsymbol{\mathcal{Y}}_1^{(i,0)}, \mathfrak{z}_i^m\right)\right)\\ &\approx \sum_{i=1}^K \log\left(\sum_{m=1}^M \widehat{p}_m^{(\ell)} \left(\sum_{\gamma=1}^{\Gamma_{\mathsf{F}}} \rho_{(i,W),m}^{(\mathsf{f},\gamma)}\right)\right)\end{aligned} \tag{S20}$$

where the last line is derived from integrating the unnormalized filtering density in Eq. (S11).

Details of the MSAR Estimation Procedure

As in the case of SSMs with GM process noise, we consider a Markov switching process in which the states are constant over consecutive windows of length W indexed by $k = 1, 2, \cdots, K$, where $K := N/W$. Recall that the parameters to be estimated are $\mathcal{M} := \left\{\{\pi_i\}_{i=1}^J, \{P_{ij}\}_{i,j=1}^{J,J}, \{\alpha_j, \boldsymbol{\mu}_j, \mathbf{Q}_j\}_{j=1}^J\right\}$. For simplicity, we assume that $\mathbf{Q}_j$ is diagonal. For notational convenience, we denote the Gaussian density with mean $\boldsymbol{\mu}$ and covariance $\mathbf{Q}$ evaluated at $\mathbf{x}$ by $\mathcal{N}(\mathbf{x}; \boldsymbol{\mu}, \mathbf{Q})$. Finally, we denote $\{\widehat{\mathbf{x}}_n\}_{n=(i-1)W+j}^{(k-1)W+l}$ by $\widehat{\mathcal{X}}_{(i,j)}^{(k,l)}$.

The parameters can be estimated using an instance of the EM algorithm as follows:

The E-step

Let $\mathcal{M}^{(\ell)} := \left\{ \{\pi_i^{(\ell)}\}_{i=1}^J, \{P_{ij}^{(\ell)}\}_{i,j=1}^{J,J}, \{\alpha_j^{(\ell)}, \boldsymbol{\mu}_j^{(\ell)}, \mathbf{Q}_j^{(\ell)}\}_{j=1}^J \right\}$ be the parameter estimates at iteration ℓ. We define:

$$\varepsilon_{j,k}^{(\ell)} := P\left[s_k = j \middle| \widehat{\mathcal{X}}_{(1,1)}^{(K,W)}, \mathcal{M}^{(\ell)} \right], \qquad \xi_{i,j,k}^{(\ell)} := P\left[s_k = j, s_{k-1} = i \middle| \{ \widehat{\mathcal{X}}_{(1,1)}^{(K,W)}, \mathcal{M}^{(\ell)} \right]. \tag{S21}$$

It can be shown that:

$$\varepsilon_{j,k}^{(\ell)} = \frac{a_{j,k}^{(\ell)} b_{j,k}^{(\ell)}}{\sum\limits_{i=1}^J a_{i,k}^{(\ell)} b_{i,k}^{(\ell)}}, \quad \xi_{i,j,k}^{(\ell)} = \frac{a_{i,k-1}^{(\ell)} P_{ij}^{(\ell)} b_{j,k}^{(\ell)} \prod\limits_{w=1}^W \mathcal{N}\big(\widehat{\mathbf{x}}_{(k,w)} - \alpha_j^{(\ell)} \widehat{\mathbf{x}}_{(k,w-1)}; \boldsymbol{\mu}_j^{(\ell)}, \mathbf{Q}_j^{(\ell)}\big)}{\sum\limits_{l=1}^J \sum\limits_{m=1}^J a_{l,k-1}^{(\ell)} P_{lm}^{(\ell)} b_{m,k}^{(\ell)} \prod\limits_{w=1}^W \mathcal{N}\big(\widehat{\mathbf{x}}_{(k,w)} - \alpha_m^{(\ell)} \widehat{\mathbf{x}}_{(k,w-1)}; \boldsymbol{\mu}_m^{(\ell)}, \mathbf{Q}_m^{(\ell)}\big)}, \tag{S22}$$

where $a_{j,k}^{(\ell)}$ and $b_{j,k}^{(\ell)}$ are the forward and backward filters, respectively, defined as:

$$a_{j,k}^{(\ell)} := P\left[s_k = j, \widehat{\mathcal{X}}_{(1,1)}^{(k,W)} \middle| \mathcal{M}^{(\ell)} \right], \qquad b_{j,k}^{(\ell)} := P\left[\widehat{\mathcal{X}}_{(k+1,1)}^{(K,W)} \middle| s_k = j, \widehat{\mathbf{x}}_{(k+1,0)}, \mathcal{M}^{(\ell)} \right]. \tag{S23}$$

The forward and backward filters can be recursively computed via the following recursions:

$$a_{j,k}^{(\ell)} = \sum_{i=1}^J a_{i,k-1}^{(\ell)} P_{ij}^{(\ell)} \prod_{w=1}^W \mathcal{N}\big(\widehat{\mathbf{x}}_{(k,w)} - \alpha_j^{(\ell)} \widehat{\mathbf{x}}_{(k,w-1)}; \boldsymbol{\mu}_j^{(\ell)}, \mathbf{Q}_j^{(\ell)}\big), \tag{S24}$$

with initialization $a_{j,1}^{(\ell)} = \pi_j^{(\ell)} \prod\limits_{w=1}^W \mathcal{N}\big(\widehat{\mathbf{x}}_{(1,w)} - \alpha_j^{(\ell)} \widehat{\mathbf{x}}_{(1,w-1)}; \boldsymbol{\mu}_j^{(\ell)}, \mathbf{Q}_j^{(\ell)}\big)$, $j = 1, 2, \cdots, J$. And,

$$b_{j,k}^{(\ell)} = \sum_{i=1}^J b_{i,k+1}^{(\ell)} P_{ji}^{(\ell)} \prod_{w=1}^W \mathcal{N}\big(\widehat{\mathbf{x}}_{(k+1,w)} - \alpha_i^{(\ell)} \widehat{\mathbf{x}}_{(k+1,w-1)}; \boldsymbol{\mu}_i^{(\ell)}, \mathbf{Q}_i^{(\ell)}\big), \tag{S25}$$

with initialization $b_{j,K}^{(\ell)} = 1$, $j = 1, 2, \cdots, J$.

Remark 3. Note that a direct implementation of the recursions in Eqs. (S24) and (S25) is likely to result in numerical instabilities, given that the Gaussian density product generates notably small values. Given that the expressions for $\varepsilon_{j,k}^{(\ell)}$ and $\xi_{i,j,k}^{(\ell)}$ are scale invariant with respect to $a_{j,k}^{(\ell)}$, $a_{j,k-1}^{(\ell)}$ and $b_{j,k}^{(\ell)}$, one can implement the recursions of Eqs. (S24) and (S25) in an *unnormalized* fashion by rescaling the forward and backward filters after each iteration as:

$$a_{j,k}^{(\ell)} \leftarrow \frac{a_{j,k}^{(\ell)}}{\sum_{i=1}^J a_{i,k}^{(\ell)}}, \qquad b_{j,k}^{(\ell)} \leftarrow \frac{b_{j,k}^{(\ell)}}{\sum_{i=1}^J b_{i,k}^{(\ell)}}. \tag{S26}$$

It is straightforward to verify that Eq. (S22) remains unchanged as a result of this rescaling procedure. The log-sum-exp approximation can also be used to further avoid numerical instabilities in computing the Gaussian density products [19].

The M-step

The Markov chain parameters can be updated as follows:

$$\pi_j^{(\ell+1)} = \frac{\varepsilon_{j,1}^{(\ell)}}{\sum\limits_{i=1}^{J} \varepsilon_{i,1}^{(\ell)}}, \qquad P_{ij}^{(\ell+1)} = \frac{\sum\limits_{k=2}^{K} \xi_{i,j,k}^{(\ell)}}{\sum\limits_{l=1}^{J}\sum\limits_{k=2}^{K} \xi_{i,l,k}^{(\ell)}}. \tag{S27}$$

The state-space parameters can be updated via coordinate descent as follows:

$$\left(\boldsymbol{\mu}_j^{(\ell+1)}\right)_m = \frac{\sum\limits_{k=1}^{K} \varepsilon_{j,k}^{(\ell)} \sum\limits_{w=1}^{W} \left((\widehat{\mathbf{x}}_{(k,w)})_m - \alpha^{(\ell)} (\widehat{\mathbf{x}}_{(k,w-1)})_m\right)}{W \sum\limits_{k=1}^{K} \varepsilon_{j,k}^{(\ell)}}, \tag{S28}$$

$$\left(\mathbf{Q}_j^{(\ell+1)}\right)_{m,m} = \frac{\sum\limits_{k=1}^{K} \varepsilon_{j,k}^{(\ell)} \sum\limits_{w=1}^{W} \left((\widehat{\mathbf{x}}_{(k,w)})_m - \alpha^{(\ell)} (\widehat{\mathbf{x}}_{(k,w-1)})_m - (\boldsymbol{\mu}_j^{(\ell+1)})_m\right)^2}{W \sum\limits_{k=1}^{K} \varepsilon_{j,k}^{(\ell)}}, \tag{S29}$$

for $m = 1, 2, \cdots, d_x$, $j = 1, 2, \cdots, J$, and

$$\alpha_j^{(\ell+1)} = \frac{\sum\limits_{k=1}^{K} \varepsilon_{j,k}^{(\ell)} \sum\limits_{m=1}^{d_x} \frac{1}{\left(\mathbf{Q}_j^{(\ell+1)}\right)_{m,m}} \sum\limits_{w=1}^{W} (\widehat{\mathbf{x}}_{(k,w-1)})_m \left((\widehat{\mathbf{x}}_{(k,w)})_m - (\boldsymbol{\mu}_j^{(\ell+1)})_m\right)}{\sum\limits_{k=1}^{K} \varepsilon_{j,k}^{(\ell)} \sum\limits_{m=1}^{d_x} \frac{1}{\left(\mathbf{Q}_j^{(\ell+1)}\right)_{m,m}} \sum\limits_{w=1}^{W} (\widehat{\mathbf{x}}_{(k,w-1)})_m^2}. \tag{S30}$$

The EM algorithm proceeds until a convergence criterion is met at iteration L_0, upon which the MSAR estimates can be computed as:

$$\widehat{\mathbf{x}}_{(k,w)}^{(\mathsf{MSAR})} := \sum_{j=1}^{J} \omega_{j,k}^{(L_0)} \left(\boldsymbol{\mu}_j^{(L_0)} + \alpha_j^{(L_0)} \widehat{\mathbf{x}}_{(k,w-1)}\right), \quad n = 1, 2, \cdots, N, \tag{S31}$$

where

$$\omega_{j,k}^{(L_0)} := P\left[s_k = j \middle| \widehat{\mathcal{X}}_{(1,1)}^{(k,W)}, \mathcal{M}^{(L_0)}\right] = \frac{a_{j,k}^{(L_0)}}{\sum_{i=1}^{J} a_{i,k}^{(L_0)}}. \tag{S32}$$

References

1. Cappé O, Godsill SJ, Moulines E. An overview of existing methods and recent advances in sequential Monte Carlo. Proceedings of the IEEE. 2007;95(5):899–924.

2. Olsson J, Cappé O, Douc R, Moulines E, et al. Sequential Monte Carlo smoothing with application to parameter estimation in nonlinear state space models. Bernoulli. 2008;14(1):155–179.

3. Klaas M, Briers M, De Freitas N, Doucet A, Maskell S, Lang D. Fast particle smoothing: If I had a million particles. In: Proceedings of the 23rd International Conference on Machine Learning. ACM; 2006. p. 481–488.

4. Fearnhead P, Wyncoll D, Tawn J. A sequential smoothing algorithm with linear computational cost. Biometrika. 2010;97(2):447–464.

5. Vo BN, Vo BT, Mahler RP. Closed-form solutions to forward–backward smoothing. IEEE Trans on Sig Proc. 2012;60(1):2–17.

6. Anderson BD, Moore JB. Optimal filtering. Courier Corporation; 2012.

7. Wan EA, Van Der Merwe R. The unscented Kalman filter for nonlinear estimation. In: Proceedings of the IEEE 2000 Adaptive Systems for Signal Processing, Communications, and Control Symposium. IEEE; 2000. p. 153–158.

8. Gibson S, Ninness B. Robust maximum-likelihood estimation of multivariable dynamic systems. Automatica. 2005;41(10):1667–1682.

9. Kitagawa G. The two-filter formula for smoothing and an implementation of the Gaussian-sum smoother. Annals of the Institute of Statistical Mathematics. 1994;46(4):605–623.

10. Wong R. Asymptotic approximations of integrals. SIAM; 2001.

11. Sorenson HW, Alspach DL. Recursive Bayesian estimation using Gaussian sums. Automatica. 1971;7(4):465–479.

12. Runnalls AR. Kullback-Leibler approach to Gaussian mixture reduction. IEEE Trans on Aero and Elec Sys. 2007;43(3):989–999.

13. Crouse DF, Willett P, Pattipati K, Svensson L. A look at Gaussian mixture reduction algorithms. In: 14th International Conference on Information Fusion. IEEE; 2011. p. 1–8.

14. Ding J, Tarokh V, Yang Y. Model selection techniques: An overview. IEEE Sig Proc Mag. 2018;35(6):16–34.

15. Figueiredo MAT, Jain AK. Unsupervised learning of finite mixture models. IEEE Trans on Patt An & Mach Int. 2002;(3):381–396.

16. Biernacki C, Celeux G, Govaert G. Assessing a mixture model for clustering with the integrated completed likelihood. IEEE Trans on Patt An & Mach Int. 2000;22(7):719–725.

17. Mehrjou A, Hosseini R, Araabi BN. Improved Bayesian information criterion for mixture model selection. Pattern Recognition Letters. 2016;69:22–27.

18. De Jong P. The likelihood for a state space model. Biometrika. 1988;75(1):165–169.

19. Boyd S, Vandenberghe L. Convex optimization. Cambridge University Press; 2004.
