# Supplementary material for: Dynamic estimation of auditory temporal response functions via state-space models with Gaussian mixture process noise
Source: PLoS Comput Biol. 2020 Aug 19;16(8):e1008172. doi: 10.1371/journal.pcbi.1008172 (PMC7485982; doi:10.1371/journal.pcbi.1008172)
Supplement: Attachment — Submitted filename: SSGM_PLoS_Response.pdf [file pcbi.1008172.s002.pdf]

UNIVERSITY OF MARYLAND

Behtash Babadi, Associate Professor
Department of Electrical & Computer Engineering
Institute for Systems Research
2351 A. V. Williams Building
University of Maryland, College Park
College Park, MD 20742
Phone: (301)-405-8346
Email: behtash@umd.edu

A. JAMES CLARK SCHOOL OF ENGINEERING
GLENN L MARTIN INSTITUTE OF TECHNOLOGY
DEPARTMENT OF ELECTRICAL & COMPUTER ENGINEERING

July 6, 2020

Dear Editorial Board of PLoS Computational Biology,

We are pleased to present for your consideration our revised manuscript titled *"Dynamic Estimation of Auditory Temporal Response Functions via State-Space Models with Gaussian Mixture Process Noise" (PCOMPBIOL-D-20-00251, submitted 02/14/2020)*. We thank the editor and reviewers for their insights and care, and based on their comments and critiques, we have now significantly revised the presentation and results of our manuscript. The major changes include:

1) Restructuring the manuscript by emphasizing the biological relevance of our work, by streamlining the results in a more accessible fashion, and by moving the technical details to the S1 Appendix, as suggested by the Editor and Reviewers 1 & 2;
2) Including another baseline method based on Markov switching autoregressive models, as suggested by Reviewer 1;
3) Including the analysis of another MEG dataset from an attention switching experiment with more subjects and trials, as suggested by Reviewer 3;
4) Incorporating various points of discussion and new references in the Introduction, Results, Methods and Discussion sections, as suggested by Reviewers 1, 2 & 3.

In what follows, we respond in detail (in black) to each of the reviewer's comments (in green). The corresponding changes are also marked in blue in the marked-up copies of the revised manuscript and S1 Appendix. We hope that you will find our revised manuscript suitable for publication in PLoS Computational Biology. We look forward to hearing from you soon.

Sincerely,
On behalf of the authors,

Behtash Babadi
Associate Professor
Department of Electrical & Computer Engineering
Institute for Systems Research
2351 A. V. Williams Building
University of Maryland, College Park
College Park, MD 20742
Phone: (301)-405-8346
Email: behtash@umd.edu

Response to the Comments of Reviewer 1

The paper introduces a Gaussian Mixture (GM) state space method (SSM) designed to estimate the latent dynamics of neural systems.

The method is particularly suitable in experimental settings were sudden switches between neural states are expected since they induces multi-modal distributions that are well-captured by GM models.

one of these settings is dichotic listening, where the participant can switch their attention towards one of two simultaneously presented speech recordings.

The core of the paper is a family of expectation-maximization EM algorithms for efficient maximum likelihood inference. The proposed algorithms cleverly are well designed and properly validated. The proposed method is definitely relevant for cognitive neuroscience as multi-modality is common in the analysis of brain signals and it can lead to meaningful interpretation of the data in terms of simpler latent signals.

In general, while I highly appreciate the technical quality of the paper, I think that at the present state it is more suitable for a machine learning/sigma processing venue. However, the paper could fit in PLOS comp bio if the authors put some effort in improving the exposition and stressing the neuroscientific motivations.

Response: We thank the reviewer for his positive assessment and suggestions for improving the presentation of the manuscript.

Major Comments:

1) I think that the structure of the paper is not very suitable to the PLOS comp bio audience. The core of the paper is purely methodological without much reference to neuroscience. The method section should ideally be placed after the results and some of the most technical details should be moved to supplementary material. The result section should begin a high level treatment of the method focused on its underlying assumptions and its connections with alternative methods commonly used in neuroscience (i.e. TRF). This will allow the less technically minded reader to understand the main results without getting bogged on technical details that are of higher interest for the machine learning and statistics audience. Emphasis should be given on the formulation and justification of the model instead of the algorithmic solution.

Response: We thank the reviewer for this organizational comment. We have now significantly reorganized the various sections in the paper to emphasize the neuroscience implications of our work and make the results more streamlined. In particular,

1) Per the reviewer's suggestion, we have now placed the Methods section (page 22) after the Results section (page 7). Also, most of the technical details of the Methods section are now moved to the Methods section (new subsection on pages 26-29 called Dynamic Estimation of the TRF) and the S1 Appendix.
2) Per the reviewer's suggestion, we have now reorganized and revised the structure of the Results section by first describing the TRF model and its neuroscience implications at a

high level, followed by discussing the existing estimation methods and motivating the need for the proposed methodology (pages 7-9).

3) Per the reviewer's suggestion, most of the details on parameter selection pertaining to the algorithms are now moved to the Methods section (new subsection on page 29 called Model Parameter Settings).

- *In response to this comment, we have now significantly revised and reorganized the presentation of the Results and Methods section to emphasize the neuroscience implications as well as to provide justification on the need for our proposed methodology.*

2) The results of the method should be compared with a wider range of baseline models. In particular, there need to be a quantitative comparison with Markov-switching autoregressive models as they are an obvious alternative to the GM approach. These models should be also discussion in the introduction.

Response: We thank the reviewer for his suggestion on including other baseline methods. We have now added smoothed TRF estimates obtained by a Markov-Switching Autoregressive (MSAR) model to the baseline methods in our revised manuscript. In particular, we have now discussed MSAR models in the Introduction (page 5, lines 70-75), Discussion (pages 21, lines 407-416), and Methods (pages 27-28, lines 570-590), and have included it in our simulation studies (pages 12-14). Given that the performance of MSAR was inferior to the other baseline methods in the simulations, we have omitted its results in our real data analysis for brevity. Also, S1 Appendix includes the details of parameter estimation for the MSAR smoothing procedure.

To the best of our knowledge, MSAR methods are mostly beneficial when the states are directly observed, and if one wants to apply an MSAR model to the states in a state-space model (with indirectly observed states), the same computational issues that we faced in our GM process noise framework would arise, i.e., intractable expectations. Thus, as a baseline method, we have applied MSAR to the least squares estimates of the states, as a surrogate of true states, which is provided by the RLS method with $\lambda = 0$.

It is noteworthy that our Gaussian mixture formulation for process noise can be considered as a special case of an MSAR state equation in a state-space model where the transition probability matrix is constrained to have equal rows. We have now stated this analogy in the Discussion and have motivated the extension to more general MSAR state-space models as a future work (page 21, lines 407-416).

- *In response to this comment, we have now added a discussion of MSAR models to the Introduction, Methods, and Discussion sections together with relevant references. We have also included it as another baseline method in the Results section.*

Minor comments:

1) Referring to w_n as noise in Eq.1 is rather confusing from a neuroscience point of view as it actually represent the cognitively modulated signal. This terminology is particularly confusing since w_n has a non vanishing expected value.

Response: We agree with the reviewer that this terminology is not ideal. We had previously discussed this issue in the Introduction, but too briefly. To avoid this confusion, we have now added text to explicitly address this in the Introduction (page 4, lines 35-46) and immediately after Eq. (1) (page 22, lines 439-446), to clarify that the *process noise* includes behaviorally- and stimulus-driven dynamics as well as the background neural activity that is not necessarily evoked by the stimulus or behavior. That said, we think adhering to this terminology is important to be consistent with the state estimation literature.

Response to the Comments of Reviewer 2

"Dynamic Estimation of Auditory Temporal Response Functions via State-Space Models with Gaussian Mixture Process Noise" by Miran and colleagues tackles the estimation of the neural dynamics underlying auditory process from MEG recordings in a cocktail party setting. The authors develop a method based on Expectation-Maximization algorithm to infer the parameters of a Gaussian mixture process noise from state-space observations. The method is applied both to simulated and real MEG data. They state that representing the process noise as a Gaussian mixture process significantly improves state estimation and the underlying dynamics. Finally, application to MEG data reveals improvements over existing techniques that estimate the Temporal Response Functions (TFR).

However the study is well introduced respect to previous literature, some reference about application of state space model to MEG data misses (see e.g. Sorrentino et al, 2009, 2014, Sommariva 2014).

Overall the article is well written and provides a lot of mathematical details about the developed algorithms, some of which could be moved to Appendix to facilitate the reading. Indeed sometime the reader could loose the final aim to estimate the parameters of the Gaussian Mixture process noise.

Response: We thank the reviewer for his/her positive assessment and suggestions for improving the presentation of the manuscript. We have now included the additional references [8]-[10] on the use of SMC methods in MEG data analysis in the Introduction (page 3, line 8 and lines 23-24). Also, in response to the organizational comment of the reviewer, as well as a similar comment by Reviewer 1, we have now moved most of the technical details to S1 Appendix.

➢ *In order to address these comments, we have now included the suggested references and moved most of the technical details to S1 Appendix.*

I have very few comments:

- line 112: "To represent the process noise" should be To represent the process noise wn

Response: Done (page 22, line 447).

- In line 126 for the sake of clarity should be better replacing n1, n2 with 1 to N

Response: Now that those technical details have been moved to S1 Appendix, we have deferred the definition using this more involved notation to S1 Appendix and only use indices 1 to N in the Methods section (page 23, lines 462-463).

- Some information on computational time on a generic machine could be added

Response: We have now added a numerical comparison of the run-time when we discuss the computational advantages over particle smoothing (page 13, lines 263-265).

- In both simulated and read data, a discussion on sensitivity to chosen parameters (e.g. M, number of particles) could be given

Response: We have now added these discussions (page 12, lines 244-249, and page 30, lines 653-658).

- An overall analysis of the drawbacks of the method could be added

Response: Thank you for this suggestion. We have now added a discussion of the limitations and drawbacks of the proposed method to the Discussion section (page 20, lines 389-397).

Response to the Comments of Reviewer 3

The authors present a state-space model in which the process noise is modeled by a mixture of Gaussians. They derive the EM updating equations, where some of the variables turn out to be intractable. The authors then provide two algorithms to estimate these: one based on Monte-Carlo and one based on approximative closed-form expressions based on Taylor approximations. The method is applied in a dynamic TRF estimation problem. In simulations, both approaches are almost equally accurate, whereas the latter is much faster in terms of computations.

The paper is interesting and very well written. I only have one major comment (and several minor ones, see below).

Response: We thank the reviewer for his positive assessment and suggestions for improving the presentation of the manuscript.

While the simulations are convincing, I find the results provided on real data a bit too anecdotal (two trials of a single subject). It would be fine as an illustrative example of a potential application for the proposed algorithm, but as the title does emphasize TRF estimation as the focus of the paper, I feel the real-data experiments are not that convincing. Would it be possible to do a group analysis and to show that the GM-based model results in a statistically significant improvement wrt RLS or a linear Gaussian SSM? I know it is hard due to lack of a ground truth TRF, but perhaps the attention decoding accuracy could be used as an indirect performance metric. A group-wise version of Fig. 6E may also be interesting (after aligning the attention switches of the different subjects), showing medians and quantiles across subjects.

Response: We thank the reviewer for his suggestion and do agree that a group-wise analysis is much more informative. We have now included a group-wise analysis over the at-will attention switching experiment as well as an instructed attention switching experiment, which was performed at the University of Maryland prior to the at-will attention switching experiment. Although 5 subjects participated in the at-will attention switching experiment, only one of the subjects (3 trials) exhibited an auditory DSS rotation matrix and had a reliable behavioral report. We agree that we need more subjects and trials for the at-will attention switching experiment, but have unfortunately been unable to collect more data due to the impact of the ongoing pandemic.

To remedy this shortcoming, we have now included data we had already recorded from an instructed attention switching experiment (7 subjects, 6 trials each) in our revised analysis (pages 13-15, 17-20, and 32-33). In this experiment, subjects were instructed to maintain attention on a speaker for the first 30 s of the trial, switch to the other speaker at the 30 s mark in the middle of a trial, and maintain attention on the latter speaker until the end of the 60 s trial. We still believe that the at-will attention switching experiment is an interesting one, and our Gaussian mixture formulation would be more beneficial to capture the quick dynamics due to multiple free attention switches. Thus, we have included the data from the one subject in the at-will attention switching experiment separately from the instructed attention switching experiment and hope more conclusive results can be produced for this challenging experiment in the future. For both data sets, we have shown the scatter/box plots for the attention decoding accuracy in Fig. 8 (page 19).

As the reviewer suggested, the attention decoding accuracy is not an ideal performance metric for TRF estimation quality, due to the absence of a ground truth, and given that the SSM with GM process noise only results in a modest increase of mean attention decoding accuracy over the Gaussian SSM. However, the AIC model selection criteria for the number of Gaussians in the GM shows that in none of the 45 trials, a Gaussian SSM is chosen over a GM state noise. We have also included the AIC model selection results in Fig. 8 and believe it provides complementary justification for using a GM process noise. These explanations have all been added to the Results (pages 13-15 and 17-20).

- *In response to this comment, we have now included the analysis of a MEG data from another attention switching experiment to our revised manuscript, have performed group-wise analyses over the old and new data, and included the AIC results as a complementary metric to mean attention decoding accuracy.*

Detail comments:

- In the introduction the authors motivate the use of GMs for the process noise, while claiming that a single Gaussian is sufficient to model measurement noise. I don't fully agree with that statement. In the case of neural signals there are often artifacts, which may result in outliers in the signals which typically do not follow Gaussian statistics. Moreover, the noise process in (30) is background MEG activity (unrelated to the stimulus response). Background MEG is known to change over time, i.e., different covariance matrices R would be 'active' in different segments of the data. So it would also make sense to model measurement noise in a similar fashion as proposed for the process noise. Can the method be extended to also include Gaussian mixtures in the observation noise? (I am not asking to include this, but rather to briefly comment on this case and how difficult/trivial it would be to add this extension).

Response: This is a great point, with which we fully agree. Given that the observed signals are directly accessible, source separation techniques are often used to remove the non-Gaussian, non-stationary, and background components of the observed data (e.g., ICA, DSS, TSPCA methods). As such, the remaining sources of uncertainty in the "denoised" data (following source separation) often admit Gaussian models. The states, however, are indirectly observable, which critically limits the usage of source separation techniques. In addition, the goal is not to "remove" the process noise, as it models the key stochastic features of the state evolution, but to model it and account for it. As a result, we have mainly considered GMs for the state-noise, assuming that the observations admit a Gaussian noise model. We have now added this discussion to the Introduction (pages 3-4, lines 25-46).

Finally, as the reviewer suggested, it is indeed possible to model the observation noise via GMs and infer its parameters jointly with the process noise within the same model. The resulting algorithms, however, are more involved. We have now added a discussion to this effect in the Discussion section (pages 20-21, lines 398-406).

➢ *In order to address this comment, we have now significantly revised the discussion of observation and process noise assumptions in the Introduction, and added a discussion on the extension of the GM inference framework to modeling the observation noise in the Discussion.*

-The SMCEM acronym is not explained in intro

Response: Defined now (page 5, lines 84-85). Thanks for catching this.

-Which bias filter was used in the DSS method? Usually repeated stimuli are required, but the description of the experiment protocol does not mention any repeated stimuli. Please elaborate.

Response: We have used the usual averaging bias filter for testing consistency across trials. As mentioned in "Subjects, Stimuli, and Procedures" subsection in Methods (page 33, lines 729-731), pilot studies were performed for each participant prior to the main attention switching experiments, where subjects listened to a single-speaker stimulus for three repeated trials. DSS rotation matrices were computed using these pilot trials. We have now reworded this section to

clarify the choice of the bias filter in the "MEG Data Preprocessing" subsection (page 33, lines 731-734).

-page 19: I guess y_t is one of the DSS components? Please explain this.

Response: That is correct. We have now explained this right after defining y_t (page 26, lines 534-537).

-it is mentioned that the parameter alpha can be estimated within the EM framework. It may be useful for the reader to have the corresponding updating equation (could be added in an appendix). In fact, it could be useful to provide all the updating equations for the model defined in (32) as a special case of the more general updating equations.

Response: This is a great suggestion. We have now added the update rule for α in Remark 1, where we discuss the update rule for the observation noise covariance **R** (page 25, lines 514-518). We have also included the explicit update rules for **R** and α in the special case in the S1 Appendix.

- it is mentioned that -alternatively- the alpha parameter can be set based on the domain-specific knowledge of the problem to provide a desired adaptivity-robustness trade-off. I find this statement a bit strange, as the state dynamics are defined by the dynamics of the underlying (neural) processes. The word 'desired' therefore seems a bit strange here. Although I do understand what the authors mean (this parameter is indeed akin to the forgetting factor in RLS), I would rephrase or elaborate a bit.

Response: We have now rephrased this sentence by replacing the word "desired" with "target", and mentioned the analogy with the forgetting factor in the RLS algorithm (page 29, lines 602-605).

-the second strategy to initialize the EM process (page 24, line375): Is this based on the oracle Gaussian fit in Fig. 2-C? Or do you mean to first run a naive model with a single Gaussian? Please explain this a bit better.

Response: Yes, we first run the Linear Gaussian SSM over the data and compute the state residuals (i.e., empirical samples of the state noise) as $\widehat{\boldsymbol{w}}_t = \widehat{\boldsymbol{x}}_t^s - \alpha \widehat{\boldsymbol{x}}_{t-1}^s$, where $\widehat{\boldsymbol{x}}_t^s$ represents the smoothed state at time t using the Linear Gaussian SSM. Then, we fit a GM on the residuals and initialize the EM algorithm with these parameters. The state residuals from the linear Gaussian SSM do not have a clear multimodal histogram due to the assumption of Gaussianity in this model and the imperfect state estimates. However, a GM fit on these samples can serve as a reasonable initialization for EM in our experience. We have now included this explanation in the subsection Model Parameter Settings in the Methods (pages 29-30, lines 627-636).

- It is mentioned that the results of Fig. 3 are based on the first initialization strategy for the EM procedure. Is that also the case for Fig. 4 and 5? Are there no results for the second initialization strategy?

Response: We thank the reviewer for bringing this to our attention. In all of the simulations, we have used the first initialization strategy to show that under reasonable SNRs, the algorithm is able to start with a large covariance (based on the estimate from the Linear Gaussian SSM) and subsequently retrieve the concentrated mixture components. This is analogous to particle smoothing methods in which the initial samples are drawn from a broad density and through weighting and resampling the particles can eventually model the mixture components. In our experience, the second strategy can result in faster convergence, especially under poor SNR conditions, due to the extra information extracted from the residual estimates in the Linear Gaussian SSM. Thus, for our real data analysis, we initialized the EM algorithms using the second method. We have now added this explanation to the "Model Parameter Settings" subsection (page 30, lines 637-646).

-Fig. 5: it would help if the ground truth TRF heatmap is also added here for convenience

Response: Done (page 14). Thanks for this suggestion.

-In the last sentence of the 'results' section, the authors provide attention decoding accuracies. How are these defined? Are they computed over the entire trial on a sample-by-sample basis?

Response: These are computed over the entire trial. In other words, whenever $M_{100}^{att} - M_{100}^{unatt}$ falls below zero in the trial, the corresponding time interval is marked as a misclassification. The total misclassification rate for a trial is just the sum of these time intervals divided by the length of the trial. We have now added this information prior to the attention decoding accuracy results for clarification (pages 16-17, lines 324-329).

- The description of the data set mentions five subjects, yet only one subject is reported in the results.

Response: As mentioned in our response to the reviewer's major comment, although 5 subjects participated in the at-will attention switching experiment, only one of the subjects (3 trials) exhibited an auditory DSS rotation matrix and had a reliable behavioral report. Therefore, we have only shown the results for that one subject. We have stated this in the subsection "Subjects, Stimuli, and Procedures" in the Methods (page 32, lines 700-702).

-Typo: Although the estimated process noise variance in in the GM case is controlled by... --> 2x 'in'

Response: Fixed (page 15, line 313). Thanks for catching this.
